# Rapid kidney function decline and increased risk of heart failure in patients with type 2 diabetes: findings from the ACCORD cohort: Rapid kidney function decline and heart failure in T2D

**DOI:** 10.1186/s12933-023-01869-6

**Published:** 2023-06-26

**Authors:** Carlos Roberto Bueno Junior, Arjola Bano, Yaling Tang, Xiuqin Sun, Alex Abate, Elizabeth Hall, Joanna Mitri, Mario Luca Morieri, Hetal Shah, Alessandro Doria

**Affiliations:** 1 Research Division, Joslin Diabetes Center, Boston, MA USA; 2 Department of Medicine, Harvard Medical School, Boston, MA USA; 3 School of Physical Education and Sport, Medical School, College of Nursing of Ribeirao Preto, University of Sao Paulo (USP), Ribeirao Preto, Sao Paulo, Brazil; 4 Institute of Social and Preventive Medicine (ISPM), University of Bern, Bern, Switzerland; 5 Department of Cardiology, Bern University Hospital, University of Bern, Bern, Switzerland; 6 Department of Endocrine and Metabolism, Peking University Shuang Hospital, Beijing, China; 7 Department of Medicine, Metabolic Disease Unit, University of Padova, University Hospital of Padova, Padova, Italy; 8 Research Division, Joslin Diabetes Center, One Joslin Place, Boston, MA 02215 USA

**Keywords:** Heart failure, Diabetic kidney disease, Type 2 diabetes, Glomerular filtration rate, Albuminuria

## Abstract

**Background:**

Impaired kidney function and albuminuria are associated with increased risk of heart failure (HF) in patients with type 2 diabetes (T2D). We investigated whether rapid kidney function decline over time is an additional determinant of increased HF risk in patients with T2D, independent of baseline kidney function, albuminuria, and other HF predictors.

**Methods:**

Included in the study were 7,539 participants in the Action to Control Cardiovascular Risk in Diabetes (ACCORD) study with baseline urinary albumin-to-creatinine ratio (UACR) data, who had completed 4 years of follow-up and had ≥ 3 eGFR measurements during that period (median eGFR/year = 1.9, IQR 1.7–3.2). The association between rapid kidney function decline (eGFR loss ≥ 5 ml/min/1.73 m^2^/year) and odds of HF hospitalization or HF death during the first 4 years of follow-up was estimated by logistic regression. The improvement in risk discrimination provided by adding rapid kidney function decline to other HF risk factors was evaluated as the increment in the area under the Receiving Operating Characteristics curve (ROC AUC) and integrated discrimination improvement (IDI).

**Results:**

Over 4 years of follow-up, 1,573 participants (20.9%) experienced rapid kidney function decline and 255 (3.4%) experienced a HF event. Rapid kidney function decline was associated with a ~ 3.2-fold increase in HF odds (3.23, 95% CI, 2.51–4.16, p < 0.0001), independent of baseline CVD history. This estimate was not attenuated by adjustment for potential confounders, including eGFR and UACR at baseline as well as at censoring (3.74; 95% CI 2.63–5.31). Adding rapid kidney function decline during follow-up to other clinical predictors (WATCH-DM score, eGFR, and UACR at study entry and end of follow-up) improved HF risk classification (ROC AUC = + 0.02, p = 0.027; relative IDI = + 38%, p < 0.0001).

**Conclusions:**

In patients with T2D, rapid kidney function decline is associated with a marked increase in HF risk, independent of starting kidney function and/or albuminuria. These findings highlight the importance of serial eGFR measurements over time to improve HF risk estimation in T2D.

**Supplementary Information:**

The online version contains supplementary material available at 10.1186/s12933-023-01869-6.

## Background

Heart failure (HF) is a frequent complication of type 2 diabetes (T2D), contributing to the excess morbidity and mortality characteristic of this disease [1–5]. Up to 50% of patients with T2D may develop HF during their lifetime [2], corresponding to a two- to five times higher HF risk than in subjects without diabetes [1, 2, 4]. The higher risk of HF experienced by patients with T2D can be a consequence of the higher prevalence of coronary artery disease [6] and hypertension [7] associated with T2D, but may also result from the development of diabetic cardiomyopathy - a diabetes-specific disease of the myocardium resulting from exposure to diabetic milieu, independent of ischemic lesions [3, 8, 9].

A major risk factor for HF among patients with T2D is the presence of diabetic kidney disease (DKD). Large epidemiological studies have shown that both clinical alterations characteristic of DKD – impaired glomerular filtration rate (GFR) and increased urinary albumin excretion – are associated with an increased risk of HF independently from each other [2, 10–12]. A few studies in the general population have suggested that a rapid rate of kidney function decline over time – the parameter that has been shown to best capture the disease processes underlying chronic kidney disease [13, 14] – is an additional risk factor for the development of HF [12, 15, 16]. However, whether and to what extent this applies to patients with diabetes, who are already at increased risk of developing kidney dysfunction and HF, has not been investigated. It remains also unknown whether the association between a rapid rate of kidney function decline and HF risk is independent of kidney function and albuminuria at baseline. Disentangling these processes underlying the link between DKD and HF can have crucial implications for improving prediction of HF and devising new strategies to prevent HF in patients with DKD.

In this study, we analyzed longitudinal data from the Action to Control Cardiovascular Risk in Diabetes (ACCORD) clinical trial to investigate the association of rapid kidney function decline over time with risk of HF in patients with T2D, and to assess whether this was independent from kidney function and albuminuria at baseline. We also assessed whether accounting for changes in eGFR over time improved prediction of the risk of HF events as compared to standard clinical predictors.

## Methods

### Study population

The aim of the Action to Control Cardiovascular Risk in Diabetes (ACCORD) clinical trial (NCT00000620) was to investigate whether cardiovascular event rates could be reduced by intensively targeting hyperglycemia to HbA1c < 6.0%, compared to a standard target of HbA1c between 7-7.9% [17, 18]. For this purpose, 10,251 participants with T2D and high cardiovascular risk were randomized in a 1:1 ratio to receive intensive or standard glycemic control therapy at 77 clinical sites across the U.S. and Canada. The study also investigated the effect of intensive versus standard blood pressure (BP) control and fibrate versus placebo therapy on cardiovascular events through the ACCORD BP and Lipid sub-trials in a double 2 by 2 factorial design [18]. Additionally, ACCORD had a rich follow-up of study participants and collected data on other diabetic complications, both at baseline and during follow-up. The full protocol for the main ACCORD trial has been previously published [18]. Inclusion criteria included (1) T2D and HbA1c ≥ 7.5%; (2) age 40–79 years and known cardiovascular disease (CVD); or (3) age 55–79 years with anatomic evidence of significant atherosclerosis, albuminuria, left ventricular hypertrophy, or at least two risk factors for CVD (dyslipidemia, hypertension, obesity, or current smoker status). The glycemia trial was stopped after a mean of 3.7 years because of the finding of excess mortality in the intensive glucose-lowering group [19]. Participants were managed according to the standard glucose protocol and monitored for an additional 17 months while the BP and lipid trials were completed. Ethics committees at each center approved the protocol, which adhered to the Declaration of Helsinki [18]. All participants provided written, informed consent. The present analysis included ACCORD participants who (i) completed 4 years of follow-up and/or experienced a HF event in that time period, (ii) had at least three values of eGFR between baseline and the HF event or the 4 year time point, from which eGFR slopes could be calculated, and (iii) had available data on urine albumin-to-creatinine ratio (UACR) at baseline (Supplementary Fig. 1).

### Exposures

Exposures of interest were the eGFR and UACR at baseline and at the last visit before the HF event or 4 year censoring and the rate of eGFR change during follow-up (expressed as ml/min/1.73 m^2^ per year). eGFR was estimated in ACCORD at baseline and at regular intervals during follow-up from serum creatinine, measured by the Roche Creatinine Plus enzymatic method (Roche Diagnostics, Basel, Switzerland), using the 2021 CKD-EPI Serum Creatinine equation [20]. For each participant, the absolute rate of eGFR change over time (“eGFR slope”) was estimated by least-squares regression of all eGFR measurements from month 4 of follow-up to the last eGFR measurement before the HF event or the 4-year censoring date (Supplementary Fig. 1). Month 4 (corresponding to the first visit after randomization) was used as the first time point to account for the fact that one of the interventions investigated in ACCORD (fenofibrate) was known to cause an increase in serum creatinine levels, which would have caused a systematic bias in the eGFR slope estimation in this treatment arm if the slope had been calculated from randomization [21]. In agreement with the Kidney Disease Improving Global Outcomes (KDIGO) 2012 guidelines [22], rapid kidney function decline was defined as a sustained eGFR decline ≥ 5 ml/min/1.73 m^2^ per year. Urine creatinine was determined enzymatically on a Roche Double Modular P Analytics automated analyzer. Urine albumin was determined by immunonephelometry on a Siemens BN II nephelometer. Albuminuria was defined as UACR ≥ 30 mg/g, with microalbuminuria defined as UACR between 30 and 299 mg/g and overt proteinuria as UACR above 300 mg/g.

### Heart failure

Heart failure was defined as HF death or hospitalization for HF [18, 19]. HF death was defined as death due to clinical, radiological or postmortem evidence of HF without clinical or postmortem evidence of an acute ischemic event. Hospitalization for HF was documented by clinical and radiological evidence and confirmed by an adjudication committee in ACCORD. HF events were queried at each study visit.

### Additional measurements

Demographic characteristics, diabetes duration, smoking status, medical and medication history were determined at the baseline visit using standardized questionnaires. Height, weight and systolic BP were measured according to a standardized protocol. HbA1c was measured by an automated high-performance liquid chromatography (Tosoh Bioscience, South San Francisco, CA). All baseline laboratory values were obtained centrally at the University of Washington Northwest Lipid Metabolism and Diabetes Research Laboratory. The WATCH-DM HF risk score was calculated from age, BMI, systolic and diastolic blood pressure (SBP and DBP), fasting plasma glucose, serum creatinine, HDL cholesterol, QRS duration on EKG, prior myocardial infarction, and prior coronary artery bypass graft classes and used to subdivide participants in 5 HF risk classes as described by Segar et al [23].

### Statistical analyses

Analyses were run in SAS v9.4 (Cary, NC). Normally distributed continuous variables were presented as mean (± standard deviation, SD) and analyzed by independent t-test for difference in means between groups. Non-normally distributed continuous variables were presented as median (inter-quartile range) values and analyzed by t-test after log transformation. Categorical variables were presented as counts (percentages) and analyzed by chi-square tests to examine differences among groups.

In order to examine the association of rapid kidney function decline with the risk of HF events, unadjusted (Model 1) and adjusted (Models 2–5) odds ratios and their 95% confidence intervals were estimated by multivariable logistic regression models. Analyses were adjusted for ACCORD clinical centers and treatment assignments (Model 2) and for potential confounders that were selected based on their association with HF and/or rapid kidney function decline in ACCORD, including age, sex, diabetes duration, BMI, waist circumference, HbA1c, SBP, DBP, HDL cholesterol, triglycerides, smoking history, CVD history, diuretic therapy, beta-blocker therapy, and renin-angiotensin blocker therapy at baseline and mean Hba1c during follow-up (Model 3).

To account for the potential influence of kidney parameters at baseline: (i) we additionally adjusted the analyses for eGFR and UACR levels (continuous variables) at baseline (Model 4); and (ii) we performed subgroup analyses across strata based on eGFR and UACR levels at baseline: (1) eGFR ≥ 90 mL/min/1.73 m^2^ and normoalbuminuria (UACR < 30 mg/g), (2) eGFR ≥ 90 mL/min/1.73 m^2^ and albuminuria (UACR ≥ 30 mg/g), (3) eGFR 60–89 mL/min/1.73 m^2^ and normoalbuminuria, (4) eGFR 60–89 mL/min/1.73 m^2^ and albuminuria, (5) eGFR < 60 mL/min/1.73 m^2^ and normoalbuminuria, and (6) eGFR < 60 mL/min/1.73 m^2^ and albuminuria. To account for the potential influence of kidney function at the end of the follow-up on our results, we additionally adjusted our analyses for the last eGFR and UACR measures before the HF event or censoring (Model 5).

The following sensitivity analyses were also carried out: (1) To account for the potential influence of CVD history, we performed stratified analyses by prevalent CVD at baseline (no vs. yes). (2) To account for the potential influence that the period over which eGFR slope was calculated could have on our results, we re-did the analysis excluding individuals whose eGFR slope spanned < 2 years, in line with previous literature [24]; (3) To account for a potential influence of follow-up time on our results, we redid the analyses extending the follow-up time to 5 years instead of 4 years.

The improvement in risk discrimination provided by rapid kidney function decline during follow up before the HF event, when added to baseline and follow-up WATCH-DM HF risk score, eGFR, and UACR, was evaluated as the area under the Receiving Operating Characteristics (ROC) curve (AUC) [25] and integrated discrimination improvement (IDI) [26] by means of logistic regression models.

## Results

### Baseline clinical characteristics of ACCORD participants with rapid kidney function decline

Included in the study were 7,539 ACCORD participants, who competed 4 years of follow-up and/or had a HF event over this time period, had available UACR data at baseline, and for whom at least three eGFR values were available in the time span between four months after randomization and the HF event or the 4-year censoring (Supplemental Fig. 2). A median of 6 eGFR values were available for each participant (IQR 5, 11) over a median of 3.3 years (IQR 2.7, 3.6), corresponding to a median of 1.9 eGFR per year (IQR 1.7–3.2) (Supplemental Fig. 3). Based on eGFR slope estimates during follow-up, 1,573 participants (20.9%) had a rate of eGFR loss ≥ 5 ml/min/1.73m^2^/year and were defined as having “rapid kidney function decline”. The median rate of eGFR loss in these subjects was − 7.5 (IQR − 10.2, -6.0) as compared to -0.8 (IQR − 2.4, 0.9) ml/min/1.73 m^2^/year in the rest of participants (n = 7,760). As shown in Table 1, participants with rapid kidney function decline had longer duration of diabetes, a higher prevalence of CVD and HF history as well as diuretic treatment at baseline, a higher waist circumference, HbA1c, systolic blood pressure, total cholesterol, triglycerides, and UACR at baseline, and a higher mean Hba1c during follow-up. With regard to the interventions tested in the ACCORD trial, they had a higher prevalence of assignment to the standard rather than the intensive arm of the glycemia trial, to the intensive rather than the standard arm of the BP sub-trial, and to the placebo rather than the fenofibrate arm of the lipid sub-trial (Table 1).

**Table 1:** Characteristics of ACCORD participants according to the occurrence of rapid kidney function decline during follow-up

Characteristic	No/slow kidney function decline (N = 5,966)	Rapid kidney function decline (N = 1,573)	P value
Female (n, %)	2,246 (37.7)	517 (39.2)	0.25
**At Baseline**			
Age (years)	62.7 ± 6.6	62.8 ± 6.5	0.70
Diabetes duration (years)	10.6 ± 7.5	11.4 ± 7.6	< 0.0001
CVD history at baseline	2,037 (34.1)	579 (36.8)	0.05
HF history at baseline	229 (3.8)	85 (5.4)	0.006
BMI (kg/m^2^)	32.2 ± 5.4	32.4 ± 5.4	0.14
Waist circumference (cm)	106.5 ± 13.5	107.6 ± 13.8	0.005
HbA1c (%)	8.27 ± 1.0	8.33 ± 1.0	0.03
Fasting glucose (mg/dL)	175 ± 52	176 ± 55	0.34
SBP (mmHg)	135.9 ± 16.1	138.8 ± 16.4	< 0.0001
DBP (mmHg)	74.9 ± 10.1	74.9 ± 10.3	0.82
Antihypertensive therapy			
Renin-angiotensin blockers (n, %)	4,095 (69.0)	1,114 (71.1)	0.11
Beta-blockers (n, %)	1,733 (29.2)	482 (30.7)	0.23
Diuretics (n, %)	2,100 (35.4)	601 (38.3)	0.03
Total cholesterol (mg/dL)	183.6 ± 39.3	186.5 ± 41.4	0.01
HDL (mg/dL)	41.7 ± 11.1	41.5 ± 12	0.41
Triglycerides (mg/dL)^†^	155 (107,225)	166 (113,248)	< 0.0001
Current smoker (n, %)	785 (13.1)	208 (13.2)	0.95
Previous smoker (n, %)	3,706 (51.7)	716 (51.9)	0.79
eGFR (ml/min/1.73 m^2^)	85.8 ± 17.3	85.1 ± 16.4	0.17
eGFR category			
eGFR ≥ 90 (n, %)	2,843 (47.9)	707 (45.1)	0.009
eGFR 60–89 (n, %)	2,510 (42.3)	728 (46.4)	
eGRF < 60 (n, %)	584 (9.8)	133 (8.5)	
UACR (g/mg)^†^	13 (7,37)	21 (9,87)	< 0.0001
UACR category			
Normoalbuminuria (n, %)	4,227(70.9)	910 (57.9)	< 0.0001
Microalbuminuria (n, %)	1,439 (24.1)	481 (30.6)	
Macroalbuminuria (n, %)	300 (5.0)	182 (11.6)	
**During follow-up**			
Average HbA1c (%)	7.15 ± 0.88	7.23 ± 0.89	0.0006
eGFR slope (ml/min/1.73 m^2^/year)^†^	-0.8 (-2.4,0.9)	-7.5 (-10.2,-6.0)	By design
Last eGFR (ml/min/1.73 m^2^)^‡^	79.2 ± 18.8	61.0 ± 16.7	By design
Last eGFR category^‡^			
eGFR ≥ 90 (n, %)	2,170 (36.4)	49 (3.1)	By design
eGFR 60–89 (n, %)	2,731 (45.8)	796 (50.6)	
eGFR < 60 (n, %)	1,065 (17.9)	728 (46.3)	
Last UACR (g/mg)^†‡^	11 (6,33)	16 (7,65)	< 0.0001
Last UACR category^‡^			
Normoalbuminuria (n, %)	4,373 (73.3)	990 (62.9)	< 0.0001
Microalbuminuria (n, %)	1,309 (21.9)	404 (25.7)	
Macroalbuminuria (n, %)	284 (4.8)	179 (11.4)	
ACCORD Glycemia trial			
Standard (n, %)	2,963 (49.7)	831 (52.8)	0.03
Intensive (n, %)	3,003 (50.3)	742 (47.2)	
ACCORD BP trial			
Standard (n, %)	1,448 (54.2)	350 (39.1)	< 0.0001
Intensive (n, %)	1,222 (45.8)	546 (60.9)	
ACCORD lipid trial			
Placebo (n, %)	1,608 (48.8)	378 (55.8)	0.0008
Fibrate (n, %)	1,688 (51.2)	299 (44.2)	

Except where noted, data are means ± SD for continuous variables and counts (%) for categorical data. †Medians (IQR). ‡Last value before HF event or censoring. Abbreviations: CVD, cardiovascular disease; HF, heart failure; BMI, body mass index; HbA1c, glycated hemoglobin; SBP, systolic blood pressure; DBP, diastolic blood pressure; LDL, low density lipoprotein; HDL, high density lipoprotein; eGFR, estimated glomerular filtration rate; UACR, urinary albumin/creatinine ratio; BP, blood pressure

### Rapid kidney function decline and risk of HF events

A total of 255 participants (3.4%) experienced a HF episode in the first 4 years of follow-up. As compared to other participants who did not experience HF over 4 years follow-up (n = 7,284), these individuals were more frequently male and were characterized by older age, longer duration of diabetes, higher baseline BMI, waist circumference, HbA1c, triglycerides, and UACR, lower baseline diastolic blood pressure, total cholesterol, HDL cholesterol, and eGFR, a more frequent history of CVD, HF, and smoking, and more frequent treatment with diuretics and beta-blockers (Table 2). They also had a significantly more negative eGFR slope (median − 3.9 [IQR-9.1,0.01] vs. -1.6 [-4.2,0.4] ml/min/1.73 m^2^/year), p = 0.0002), resulting in a higher prevalence of rapid kidney function decline during follow-up (45% vs. 20%, p < 0.0001) (Table 2 and Supplementary Table 1). In an unadjusted logistic regression model, rapid kidney function decline was associated with a 3.2-fold increase in the odds of a HF episode in the first 4 years of follow-up (OR = 3.23, 95% CI, 2.51–4.16) (Table 3). Similar odds ratio estimates were obtained after stratifying the analysis by CVD history at baseline (Table 3 and Supplementary Table 1) or by limiting it to participants with a negative HF history at study entry (n = 4,535, 241 events, OR 3.66, 95% CI 2.79–4.79) or to those who did not have a history of coronary heart disease (CHD, defined as myocardial infarction, angina, and/or revascularization procedures) at study entry and did not experience CHD events at any time during follow-up (n = 4,355, 77 HF events, OR = 2.58, 95% CI 1.62–4.12), or to those whose eGFR slope estimate spanned more than two years (n = 7,177, 103 HF events, OR = 2.42, 95% CI 1.61–3.62). Results also remained similar after adjustment for ACCORD trial treatment assignments (Model 2) and multiple potential confounders (Model 3) (Table 3 and Supplementary Table 2). A similar association between rapid kidney function decline and increased HF risk was observed when analyses were conducted according to a 5- rather than 4-year time horizon (Supplementary Table 3).

**Table 2:** Characteristics of ACCORD participants according to the occurrence of heart failure during follow-up

Characteristic	No Heart Failure (N = 7,284)	Heart failure (N = 255)	P value
Female (n, %)	2,781 (38.2)	82 (32.2)	0.05
**At Baseline**			
Age (years)	62.6 ± 6.6	65.3 ± 7.2	< 0.0001
Diabetes duration (years)	10.6 ± 7.4	13.6 ± 8.7	< 0.0001
CVD history at baseline	2,466 (33.9)	150 (58.8)	< 0.0001
HF history at baseline	259 (3.6)	66 (21.6)	< 0.0001
BMI (kg/m^2^)	32.2 ± 5.4	33.8 ± 5.7	< 0.0001
Waist circumference (cm)	106.5 ± 13.5	112.3 ± 14.6	< 0.0001
HbA1c (%)	8.28 ± 1	8.45 ± 1	0.0008
Fasting glucose (mg/dL)	175 ± 53	176 ± 61	0.86
SBP (mmHg)	136.5 ± 16.2	137.1 ± 18.0	0.57
DBP (mmHg)	75.0 ± 10.1	71.2 ± 11.1	< 0.0001
Antihypertensive therapy			
Renin-angiotensin blockers (n, %)	5,020 (69.2)	189 (74.4)	0.08
Beta-blockers (n, %)	2,092 (28.9)	123 (48.4)	< 0.0001
Diuretics (n, %	2,560 (35.3)	141 (55.5)	< 0.0001
Total cholesterol (mg/dL)	184.4 ± 39.7	179.5 ± 41.1	0.05
LDL (mg/dL)	105.9 ± 32.9	102.5 ± 31.3	0.10
HDL (mg/dL)	41.8 ± 11.1	39.0 ± 9.9	< 0.0001
Triglycerides (mg/dL) Ɨ	157 (108,229)	170 (113–249)	0.13
Current smoker (n, %)	960 (13.2)	33 (12.9)	0.91
Previous smoker (n, %)	3,285 (51.4)	137 (61.2)	0.004
eGFR (ml/min/1.73 m^2^)	85.9 ± 17.0	79.3 ± 18.7	< 0.0001
eGFR category			
eGFR ≥ 90 (n, %)	3,461 (47.7)	89 (35.0)	< 0.0001
eGFR 60–89 (n, %)	3,120 (43.0)	118 (46.5)	
eGRF < 60 (n, %)	670 (9.2)	47 (18.5)	
UACR (g/mg)^†^	14 (7,41)	48 (14,224)	< 0.0001
UACR category			
Normoalbuminuria (n, %)	5,038 (69.2)	99 (38.8)	< 0.0001
Microalbuminuria (n, %)	1,811 (24.9)	109 (42.8)	
Macroalbuminuria (n, %)	435 (6.0)	47 (18.4)	
**During follow-up**			
Average HbA1c (%)	7.16 ± 0.88	7.25 ± 0.88	0.12
eGFR slope (ml/min/1.73 m^2^/year)^†^	-1.6 (-4.2,0.0.4)	-3.9 (-9.1,0.1)	0.0002
Rapid kidney function loss (n, %)	1,459 (20.0)	114 (44.7)	< 0.0001
Last eGFR (ml/min/1.73 m^2^)^‡^	75.7 ± 19.7	67.3 ± 21.0	< 0.0001
Last eGFR category^‡^			
eGFR ≥ 90 (n, %)	2,177 (29.9)	42 (16.5)	< 0.0001
eGFR 60–89 (n, %)	3,406 (46.8)	121 (47.4)	
eGFR < 60 (n, %)	1,701 (23.3)	92 (36.1)	
Last UACR (g/mg)^‡^	12 (6,35)	68 (13,220)	< 0.0001
Last UACR category^‡^			
Normoalbuminuria (n, %)	5,258 (72.2)	105 (41.2)	< 0.0001
Microalbuminuria (n, %)	1,615 (22.2)	98 (38.4)	
Macroalbuminuria (n, %)	411 (5.6)	52 (20.4)	
ACCORD Glycemia trial			
Standard (n, %)	3,760 (50.4)	124 (48.6)	0.58
Intensive (n, %)	3,614 (49.6)	131 (51.4)	
ACCORD BP trial			
Standard (n, %)	1,744 (50.4)	54 (50.9)	0.91
Intensive (n, %)	1,716 (49.6)	52 (49.1)	
ACCORD lipid trial			
Placebo (n, %)	1,903 (49.8)	83 (55.7)	0.15
Fibrate (n, %)	1,921 (50.2)	66 (44.3)	

Except where noted, data are means ± SD for continuous variables and counts (%) for categorical data. †Medians (IQR). ‡Last value before HF event or censoring. Abbreviations: CVD, cardiovascular disease; HF, heart failure; BMI, body mass index; HbA1c, glycated hemoglobin; SBP, systolic blood pressure; DBP, diastolic blood pressure; LDL, low density lipoprotein; HDL, high density lipoprotein; eGFR, estimated glomerular filtration rate; UACR, urinary albumin/creatinine ratio; BP, blood pressure

**Table 3:** Association of rapid kidney function decline during follow-up (vs. no/slow kidney function decline) with odds of heart failure within 4 years from baseline

	All Participants		No CVD History		CVD History	
Models	OR (95% CI)	P value	OR (95% CI)	P value	OR (95% CI)	P value
**Model 1**: Rapid kidney function decline	3.23 (2.51–4.16)	< 0.0001	2.82 (1.90–4.19)	< 0.0001	3.46 (2.48–4.84)	< 0.0001
**Model 2**: Model 1 **+** ACCORD trial treatment assignments and ACCORD clinical centers	3.39 (2.62–4.39)	< 0.0001	2.93 (1.96–4.39)	< 0.0001	3.59 (2.55–5.06)	< 0.0001
**Model 3**: Model 2 + sex, age, duration of diabetes, BMI, WC, baseline HbA1c, SBP, DBP, HDL, log triglycerides, smoking history, diuretic therapy, beta blocker therapy, RASB therapy, CVD history at baseline (All participants), mean Hba1c during follow-up	3.27 (2.44–4.37)	< 0.0001	2.50 (1.59–3.91)	< 0.0001	3.97 (2.69–5.87)	< 0.0001
**Model 4**: Model 3 + eGFR and log UACR at baseline	2.81 (2.08–3.78)	< 0.0001	2.24 (1.42–3.53)	0.0005	3.37 (2.25–5.06)	< 0.0001
**Model 5**: Model 4 + last eGFR and log UACR before censoring or event	3.74 (2.63–5.31)	< 0.0001	2.76 (1.61–4.73)	0.0002	4.96 (3.07–8.02)	< 0.0001

Abbreviations: OR, odds ratio; 95% CI, 95% confidence interval; BMI, body mass index; WC, waist circumference; HbA1c, glycated hemoglobin; SBP, systolic blood pressure; RASB, renin-angiotensin blockers; CVD, cardiovascular disease; eGFR, estimated glomerular filtration rate; UACR, urine albumin-creatinine ratio

*Association of rapid kidney function decline with HF risk in relation to kidney parameters at baseline.*

**Fig. 1:**
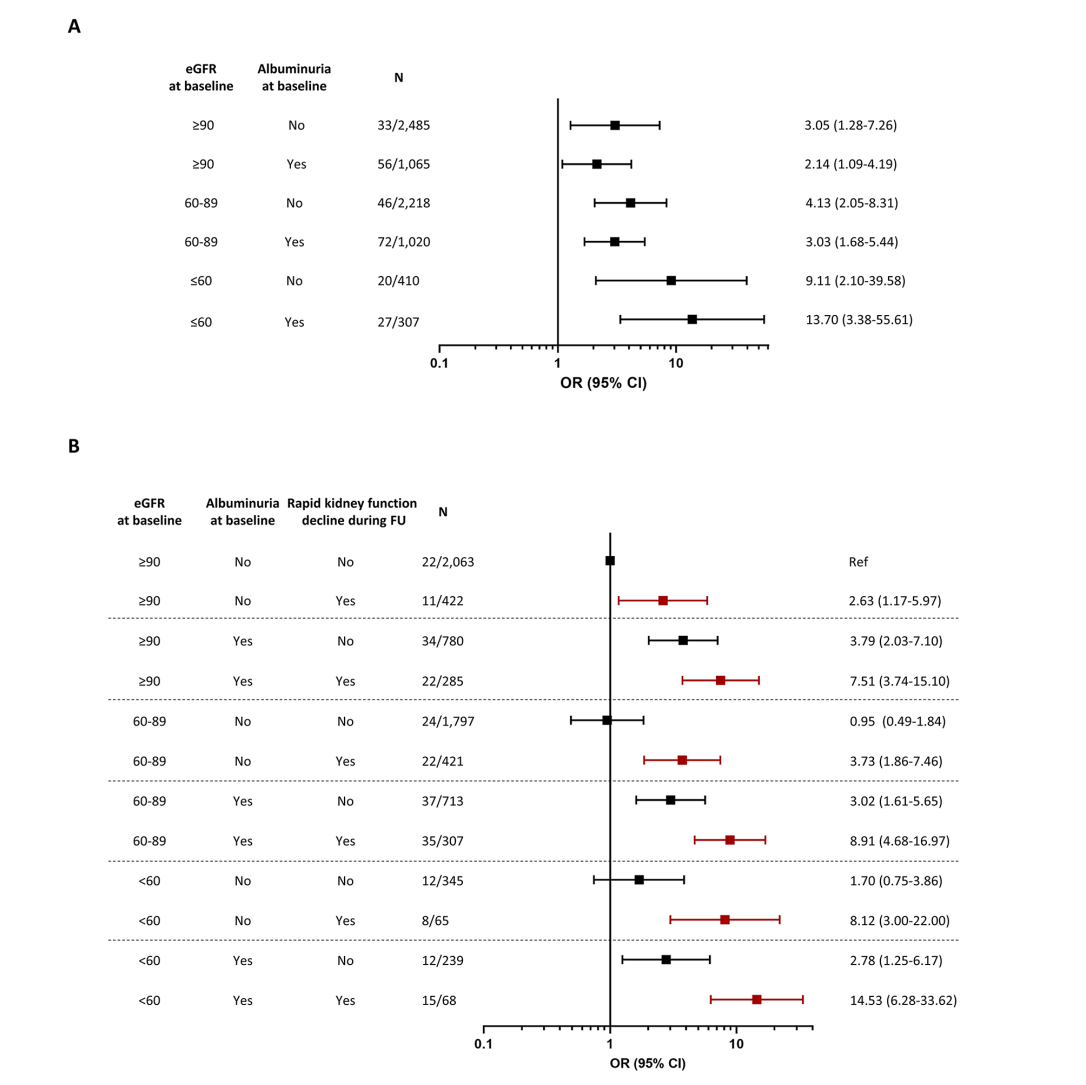
Rapid kidney function decline and risk of HF events by kidney function characteristics at baseline **(A)** Adjusted odds ratios of HF and 95% CI for rapid vs. slow/no kidney function decline in subgroups defined by eGFR and albuminuria at study entry. **(B)** Adjusted odds ratios of HF and 95% CI in subgroups defined by eGFR and albuminuria at study entry and rate of kidney function decline during follow-up. Red symbols indicate participants with rapid kidney function decline, black symbols indicate all other participants. Abbreviations: eGFR, estimated glomerular filtration rate; OR, odds ratio; CI, confidence interval; FU, follow-up

Further adjustment for eGFR and UACR at baseline, considered as continuous variables (Model 4), did not substantially change the association between rapid kidney function decline during follow-up and odds of HF (Model 4, Table 3 and Supplementary Table 2). A similar independence from baseline kidney parameters was observed in stratified analyses using eGFR and UACR categories based on accepted cut-offs of kidney function (eGFR ≥ 90, eGFR 60–89, and eGFR < 60 ml/min per 1.73 m^2^) and albuminuria (UACR ≥ 30 vs. UACR < 30 mg/g, respectively). The odds of HF were the lowest for participants with eGFR ≥ 90 or 60–89 and normoalbuminuria, highest for those with eGFR < 60 and albuminuria, and intermediate for the remaining groups (Supplementary Table 4). As shown in Fig. 1A, in all six eGFR/UACR strata, rapid kidney function decline was associated with increased odds of HF as compared to no/slow kidney function decline, without significant evidence of heterogeneity among groups (p for interaction = 0.47). As shown in Fig. 1B, participants with baseline eGFR < 60 ml/min/1.73 m^2^, increased baseline UACR, and rapid kidney function decline during follow-up had a 15-fold increase in the odds of a HF event as compared to those with none of these risk factors (OR 14.53; 95% CI 6.28–33.62). Greater than 7-fold increases in the odds of HF were also observed among participants who had experienced a rapid GFR decline and had albuminuria or an eGFR < 60.

*Association of rapid kidney function decline with HF risk in relation to kidney function at censoring.*

To determine whether the increase in HF odds observed among participants with rapid kidney function decline was an intrinsic characteristic of these individuals, or was simply due to the lower eGFR attained during follow-up because of the rapid kidney function decline or to the worsening of albuminuria, further analyses were conducted accounting for the last eGFR and UACR before the HF event or censoring (Table 1). Adjustment for these variables (Model 5, Table 3) did not attenuate the association of rapid kidney function decline with HF risk (OR, 3.74; 95% CI 2.63–5.31).

### HF risk discrimination provided by rapid kidney function decline

**Fig. 2:**
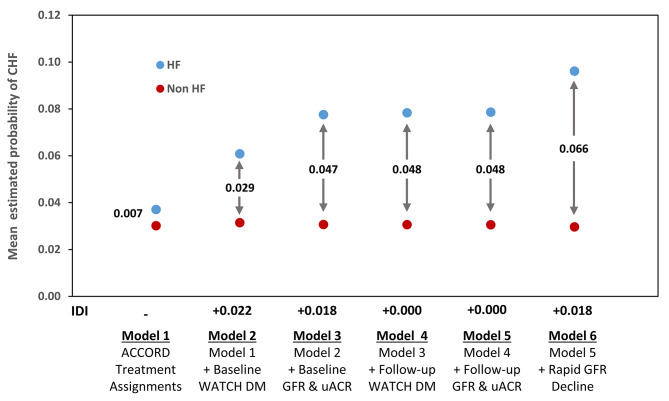
Mean estimated probabilities of HF in ACCORD participants with and without a HF event as estimated by predictive models of increasing complexities. Blue symbols represent the mean estimated probabilities of HF among participants who experienced a HF event during follow-up; red symbols represent the mean estimated probabilities among participants who did not experience a HF event. The numbers between the arrows are the differences between estimated probabilities in participants who experienced a HF event and those who did not (also known as “discrimination slopes”). The integrated discrimination improvement (IDI, equal to the difference between discrimination slopes) is reported for each model with respect to the preceding one. Abbreviations: HF, heart failure; IDI, integrated discrimination improvement; eGFR, estimated glomerular filtration rate; UACR, urinary albumin-to-creatinine ratio

The improvement in discrimination between individuals at high and low risk of HF within a 4 year timeframe provided by rapid kidney function decline was evaluated in relation to a clinical model including ACCORD trial treatment assignments, baseline and follow-up WATCH-DM HF risk score (including information on classes of age, BMI, SBP, DBP, fasting plasma glucose, serum creatinine, HDL cholesterol, QRS duration, prior myocardial infarction, and prior coronary artery bypass graft) [23] and baseline and follow-up eGFR and uACR. In terms of ROC curve [25], addition of rapid kidney function decline significantly increased the AUC from 0.77 to 0.79 (p = 0.027). If evaluated in terms of IDI [26], rapid kidney function decline increased HF risk discrimination by almost 40% (relative integrated discrimination improvement [rIDI] = 0.382, p < 0.0001). The improvement in predictive ability provided by rapid kidney function decline, as compared to that provided by WATCH-DM score, eGFR, and uACR, can be appreciated visually in Fig. 2, in which the mean probabilities of HF estimated by models of increasing complexity among subjects who experienced a HF event are plotted along the corresponding probabilities among subjects who did not. The difference between HF and non-HF, indicated by the numbers between arrows, is the so called ‘discrimination slope’ [27] and the IDI of one model vs. another model corresponds to the difference in discrimination slopes between the two models [26]. WATCH-DM score significantly improved risk discrimination (IDI = 0.022, p < 0.0001) as compared to a basic model including ACCORD treatment assignment and a further improvement (IDI = 0.018, p < 0.0001) was obtained with eGFR/uACR at baseline. Addition of the WATCH-DM score and eGFR/uACR measured immediately before the HF event or 4-year censoring did not improve risk discrimination beyond what was obtained with their baseline measures. By contrast, addition of rapid kidney function decline during follow-up (up to the HF event or censoring) resulted in a significant IDI (p < 0.0001), which was identical in magnitude (0.018) to that provided by the baseline eGFR and uACR.

## Discussion

In patients with T2D followed for 4 years, rapid kidney function decline (i.e., an eGFR decline ≥ 5 ml/min per 1.73 m^2^ per year) was associated with a 3.2-fold increase in the risk of HF as compared to no or slow kidney function decline, irrespective of cardiovascular risk factors, glycemic control, and history of CVD. The association of rapid kidney function decline with HF risk was also independent of kidney function and albuminuria at baseline. When an indicator of rapid kidney function decline during follow-up was added to these baseline characteristics and the other HF predictors captured by the WATCH-DM score, it significantly improved HF risk discrimination.

A limited number of studies have investigated the association between rate of eGFR changes and HF risk in the general population, without focusing on patients with diabetes [12, 16]. The Strong Heart Study, including 2,035 adults from the general population, reported a 1.7-fold increase in the risk of HF among participants with rapid kidney function decline over time (defined as loss of > 20 ml/min between visits) compared with the other participants [12]. The Cardiovascular Health Study, including a community-based cohort of 4,378 older individuals, reported that rapid decline in kidney function (defined as > 3 ml/min per 1.73 m^2^ per year) was associated with a 1.4-fold increase in the risk of HF [16]. These reports from the general population are extended by our study, which evaluated the association of rapid kidney function decline with HF in patients with T2D. At variance with the Strong Heart Study and Cardiovascular Heart Study, which included multiple cardiovascular outcomes,[12, 16] our analysis was focused exclusively on HF. This allowed us to perform a variety of ad hoc sensitivity analyses and account for additional factors, including glycemic control, lipid levels, CVD history, albuminuria, and eGFR at baseline and at the end of follow-up. In all these analyses, we consistently found a stronger association between rapid kidney function decline and HF, as compared with the estimates reported in the Strong Heart Study and Cardiovascular Heart Study. This could suggest that rapid kidney function decline is more predictive of HF risk in patients with diabetes than in the general population – a hypothesis that will have to be tested in specifically designed studies.

One notable feature of our findings is that the association of rapid kidney function decline with HF risk was independent of eGFR levels and albuminuria at baseline. Interestingly, it was also independent of the last eGFR measured before HF or the end of follow-up, which, as expected, was much lower in patients with rapid kidney function decline than in all other participants (61.0 vs. 79.2 ml/min/1.73 m^2^). This suggests that the increased risk of HF was related to the *rapid rate* of kidney function decline rather than the lower kidney function per se. In other words, among patients with the same degree of impairment of kidney function during follow-up, those who experienced such decline over a shorter period of time were more likely to have HF as compared to those in whom kidney function declined more slowly. Thus, evaluating kidney function and albuminuria at any given point in time may not be sufficient for patients with T2D since additional crucial information may be provided by monitoring the eGFR trajectory over time.

The increased risk of HF in patients with rapid kidney function decline over time can have several explanations. On the one hand, it is conceivable that the rapid decline of kidney function contributes to the development of HF due to the effects of a reduced kidney function on fluid retention, blood pressure regulation, and ventricular remodeling. On the other hand, since the effect of rapid kidney function decline was independent of the eGFR level reached at the end of follow-up, the association may also stem from rapid kidney function decline and HF sharing common etiological factors. In patients with diabetes, increased levels of blood pressure, dyslipidemia, and poor glycemic control have been independently associated with both the progression of renal damage and the development of HF [4, 28–32]. However, the association between rapid kidney function decline and HF observed in our study did not materially change after accounting for these shared factors. This may indicate the involvement of alternative pathways such as the renin angiotensin system, inflammation, oxidative stress, and fibrosis, whose activation is involved in both kidney disease progression and HF [33–37]. In addition, cardiac autonomic neuropathy in patients with diabetes has also been associated with an increased likelihood of both rapid kidney function decline and development of left ventricular dysfunction [38–41]. Finally, since the direction of the association between rapid kidney function decline and HF cannot be inferred from our data, it is also possible that rapid kidney function decline is the result and a subclinical manifestation of the progressive deterioration of cardiac function that will later culminate in an overt HF event. Future studies are clearly needed to infer causality and elucidate the exact mechanisms underlying the link between rapid kidney function decline and HF risk in patients with diabetes.

Strengths of the study include the large sample size, the repeated measures of kidney function over time, the detailed information on potential confounders, and the blinded adjudication of HF events. In addition, multiple sensitivity analyses provided consistent findings, increasing the confidence in these results. However, a few limitations of our study warrant consideration. The ACCORD trial included participants with T2D at high cardiovascular risk who were treated according to the standard of care at the time of the trial (between 2001 and 2009). Therefore, the generalizability of our findings needs to be confirmed in patients with diabetes who are at lower cardiovascular risk and/or are treated with newly developed glucose lowering drugs such as GLP-1 receptor agonists and SGLT2 inhibitors. Furthermore, data were not available on the different clinical presentations of HF (preserved vs. reduced ejection fraction), which may have different etiologies and predictors. Lastly, as in any observational study, the possibility of residual confounding cannot be entirely ruled out.

## Conclusions

In summary, in this analysis of data from the ACCORD clinical trial, we found that rapid kidney function decline over time was independently associated with the risk of subsequent HF in patients with T2D. These results strongly suggest that close monitoring of the eGFR trajectory over time may improve prediction and, therefore, prevention of HF in patients with diabetes, beyond what can be provided by considering individual eGFR measurements. Using rapid kidney function decline may help the early identification patients who could especially benefit from preventing treatment such as SGLT2 inhibitors – a class of drugs that has recently been found to have remarkable kidney and cardiovascular protective properties [42] Monitoring eGFR changes and starting SGLT2 inhibitors as soon as the rate of kidney function decline exceeds 5 ml/min/1.73 m^2^ per year, even in the presence of eGFR over 60 ml/min/1.73 m^2^ and normoalbuminuria, may be a cost-effective strategy to both halt further decline in renal function and prevent the development of HF.

## Electronic supplementary material

Below is the link to the electronic supplementary material.

Supplementary Material 1

## Data Availability

The ACCORD dataset is available upon request from the National Heart, Lung, and Blood. Institute Biologic Specimen and Data Repository (https://biolincc.nhlbi.nih.gov/studies/accord/).
