# Supplementary material for: Rapid kidney function decline and increased risk of heart failure in patients with type 2 diabetes: findings from the ACCORD cohort: Rapid kidney function decline and heart failure in T2D
Source: Cardiovasc Diabetol. 2023 Jun 26;22:131. doi: 10.1186/s12933-023-01869-6 (PMC10291814; doi:10.1186/s12933-023-01869-6)
Supplement: Supplementary file 1 — Supplementary Material 1 [file 12933_2023_1869_MOESM1_ESM.docx]

**SUPPLEMENTARY MATERIAL**

**Rapid kidney function decline and increased risk of heart failure in patients with type 2 diabetes: findings from the ACCORD cohort.**

***Short Title: Rapid kidney function decline and heart failure in T2D***

Carlos Roberto Bueno Junior^1,2,3^*, Arjola Bano^1,2,4,5^*, Yaling Tang^1,2^, Xuqin Sun^1,2,6^, Alex Abate^1^, Elizabeth Hall^1,2^, Joanna Mitri^1,2^, Mario Luca Morieri^7^, Hetal Shah^1,2^, Alessandro Doria^1,2^

*These authors contributed equally to this study.

^1^Research Division, Joslin Diabetes Center, Boston, Massachusetts, USA

^2^Department of Medicine, Harvard Medical School, Boston, Massachusetts, USA

^3^ School of Physical Education and Sport, Medical School, and College of Nursing of Ribeirao Preto, University of Sao Paulo (USP), Ribeirao Preto, Sao Paulo, Brazil

^4^Institute of Social and Preventive Medicine (ISPM), University of Bern, Bern, Switzerland

^5^Department of Cardiology, Bern University Hospital, University of Bern, Bern, Switzerland

^6^Department of Endocrine and Metabolism, Anzhen Hospital Affiliated to Capital Medical University, Beijing, China

^7^Department of Medicine, University of Padova, and Metabolic Disease Unit, University Hospital of Padova, Padova, Italy.

**Corresponding Author:**

Alessandro Doria, M.D., Ph.D., M.P.H.,

Research Division,

Joslin Diabetes Center,

One Joslin Place,

Boston, MA 02215

**Contents**

[**Supplementary Table 1. Prevalence of rapid kidney function decline according to CVD history at baseline and occurrence of HF events during the first 4 years of follow-up.** 3](#_Toc135312041)

[**Supplementary Table 2. Association of rapid kidney function decline and other clinical characteristics with odds of HF within 4 years from baseline.** 4](#_Toc135312042)

[**Supplementary Table 3. Association of rapid kidney function decline during follow-up (vs no/slow kidney function decline) with odds of HF within 5 years from baseline.** 5](#_Toc135312043)

[**Supplementary Table 4. Odds of HF according to baseline eGFR and UACR classes.** 6](#_Toc135312044)

[**Supplemental Figure 1. Measurements performed at baseline and follow-up.** 7](#_Toc135312045)

[**Supplemental Figure 2. Flow chart for the selection of study participants** 8](#_Toc135312046)

[**Supplemental Figure 3. Distributions of the number of eGFR measurements (A) and years spanned by them (B)** 9](#_Toc135312047)

| **Supplementary Table 1. Prevalence of rapid kidney function decline according to CVD history at baseline and occurrence of HF events during the first 4 years of follow-up.** | | | | | | | | | |
| --- | --- | --- | --- | --- | --- | --- | --- | --- | --- |
|  | **All Participants** | | | **No CVD History** | | | **CVD History** | | |
|  | **All** | **No HF** | **HF** | **All** | **No HF** | **HF** | **All** | **No HF** | **HF** |
| **No/slow kidney function decline (n [%])** | 5,966 (79.1) | 5,825 (80.0) | 141  (55.3) | 3,929 (79.8) | 3,867 (80.3) | 62  (59.1) | 2037  (77.9) | 1958 (79.4) | 79  (52.7) |
| **Rapid kidney function decline (n [%])** | 1,573 (20.9) | 1,459 (20.0) | 114  (44.7) | 994  (20.2) | 951  (19.7) | 43  (40.9) | 579  (22.1) | 508  (20.6) | 71  (47.3) |
| **All (n [%])** | 7,539 (100) | 7,284 (100) | 255  (100) | 4,923 (100) | 4,818 (100) | 105  (100) | 2,616  (100) | 2,466 (100) | 150  (100) |
| Abbreviations: HF, heart failure, CVD cardiovascular disease | | | | | | | | | |

| **Supplementary Table 2. Association of rapid kidney function decline and other clinical characteristics with odds of HF within 4 years from baseline.** | | | | | | | | | | | | | | | | |
| --- | --- | --- | --- | --- | --- | --- | --- | --- | --- | --- | --- | --- | --- | --- | --- | --- |
|  | **Model 1**  Rapid kidney function decline | | | **Model 2**  Model 1 + ACCORD trial assignments and ACCORD clinical centers | | | **Model 3**  Model 2 + sex, age, duration of diabetes, BMI, WC, HbA1c, SBP, DBP, HDL, log triglycerides, smoking history, CVD history at baseline (All participants) | | | **Model 4**  Model 3 + eGFR and log UACR at baseline | | | **Model 5**  Model 4 + eGFR and log UACR before event or at censoring | | | |
| **Predictors** | OR | 95% CI | p-value | OR | 95% CI | p-value | OR | 95% CI | p-value | OR | 95% CI | p-value | OR | 95% CI | p-value |  |
| Rapid kidney function decline | 3.23 | 2.51-4.16 | <0.0001 | 3.39 | 2.62-4.39 | <0.0001 | 3.27 | 2.44-4.37 | <0.0001 | 2.81 | 2.08-3.78 | <0.0001 | 3.73 | 2.63-5.31 | <0.0001 |  |
| Intensive glycemic control | - | - | - | 1.12 | 0.87-1.44 | 0.39 | 1.27 | 0.89-1.82 | 0.19 | 1.21 | 0.85-1.74 | 0.29 | 1.24 | 0.867-1.78 | 0.24 |  |
| Intensive BP control | - | - | - | 0.83 | 0.56-1.23 | 0.34 | 0.95 | 0.61-1.48 | 0.82 | 1.00 | 0.64-1.57 | 0.99 | 1.17 | 0.74-1.85 | 0.50 |  |
| Fenofibrate vs. placebo | - | - | - | 0.84 | 0.60-1.18 | 0.31 | 1.10 | 0.76-1.59 | 0.61 | 1.15 | 0.79-1.67 | 0.47 | 1.44 | 0.97-2.12 | 0.07 |  |
| Female | - | - | - | - | - | - | 0.98 | 0.66-1.45 | 0.92 | 1.05 | 0.70-1.56 | 0.81 | 1.09 | 0.73-1.63 | 0.67 |  |
| Age | - | - | - | - | - | - | 1.06 | 1.03-1.08 | <0.0001 | 1.06 | 1.03-1.09 | <0.0001 | 1.06 | 1.04-1.09 | <0.0001 |  |
| Diabetes duration | - | - | - | - | - | - | 1.02 | 1.00-1.04 | 0.03 | 1.01 | 0.99-1.03 | 0.21 | 1.01 | 0.99-1.09 | 0.18 |  |
| BMI | - | - | - | - | - | - | 1.03 | 0.98-1.08 | 0.29 | 1.03 | 0.98-1.08 | 0.29 | 1.03 | 0.98-1.09 | 0.26 |  |
| WC | - | - | - | - | - | - | 1.02 | 1.00-1.04 | 0.05 | 1.02 | 1.00-1.04 | 0.08 | 1.02 | 1.00-1.04 | 0.11 |  |
| Baseline HbA1c | - | - | - | - | - | - | 1.24 | 1.06-1.44 | 0.006 | 1.19 | 1.02-1.37 | 0.03 | 1.19 | 1.02-1.39 | 0.03 |  |
| Mean HbA1c during follow-up | - | - | - | - | - | - | 1.16 | 0.93-1.43 | 0.19 | 1.10 | 0.89-1.37 | 0.38 | 1.08 | 0.87-1.34 | 0.49 |  |
| SBP | - | - | - | - | - | - | 1.01 | 1.00-1.02 | 0.08 | 1.00 | 0.99-1.01 | 0.48 | 1.00 | 0.99-1.01 | 0.71 |  |
| DBP | - | - | - | - | - | - | 0.97 | 0.95-0.99 | 0.0009 | 0.98 | 0.96-0.99 | 0.006 | 0.99 | 0.96-0.99 | 0.006 |  |
| HDL | - | - | - | - | - | - | 0.99 | 0.97-1.01 | 0.40 | 0.99 | 0.97-1.01 | 0.41 | 0.99 | 0.97-1.01 | 0.39 |  |
| Log Triglycerides | - | - | - | - | - | - | 1.07 | 0.87-1.45 | 0.66 | 0.98 | 0.72-1.33 | 0.88 | 0.97 | 0.71-1.33 | 0.86 |  |
| Smoking history | - | - | - | - | - | - | 1.17 | 0.87-1.59 | 0.30 | 1.13 | 0.83-1.53 | 0.43 | 1.14 | 0.84-1.55 | 0.40 |  |
| CVD history at baseline | - | - | - | - | - | - | 1.96 | 1.44-2.67 | <0.0001 | 1.87 | 1.36-2.56 | <0.0001 | 1.84 | 1.34-2.53 | 0.0002 |  |
| Diuretic therapy at baseline | - | - | - | - | - | - | 1.59 | 2.28-2.14 | 0.002 | 1.56 | 1.15-2.11 | 0.005 | 1.53 | 1.13-2.08 | 0.006 |  |
| Beta blocker therapy at baseline | - | - | - | - | - | - | 1.27 | 0.93-1.73 | 0.13 | 1.25 | 0.92-1.71 | 0.16 | 1.28 | 0.93-1.75 | 0.13 |  |
| RASB therapy at baseline | - | - | - | - | - | - | 1.03 | 0.74-1.43 | 0.87 | 0.97 | 0.69-1.36 | 0.85 | 0.98 | 0.70-1.37 | 0.89 |  |
| eGFR at baseline† | - | - | - | - | - | - | - | - | - | 0.99 | 0.99-1.01 | 0.50 | 0.99 | 0.97-0.99 | 0.01 |  |
| Log UACR at baseline‡ | - | - | - | - | - | - | - | - | - | 1.43 | 1.31-1.57 | <0.0001 | 1.28 | 1.12-1.47 | 0.0003 |  |
| eGFR at event/censoring | - | - | - | - | - | - | - | - | - | - | - | - | 1.02 | 1.01-1.03 | 0.0008 |  |
| Log UACR at event/censoring | - | - | - | - | - | - | - | - | - | - | - | - | 1.20 | 1.06-1.35 | 0.004 |  |
| †ORs are per 10 ml/min/1.73 m2 increment in eGFR  ‡ORs are per 1 natural log mg/g increment in UACR  Abbreviations: HF, heart failure; BMI, body mass index; WC, waist circumference; HbA1c, glycated hemoglobin; SBP, systolic blood pressure; DBP, diastolic blood pressure; LDL, low density lipoprotein; HDL, high density lipoprotein; CVD, cardiovascular disease; eGFR, estimated glomerular filtration rate; UACR, urinary albumin/creatinine ratio. | | | | | | | | | | | | | | | | |

| **Supplementary Table 3. Association of rapid kidney function decline during follow-up (vs no/slow kidney function decline) with odds of HF within 5 years from baseline.** | | | | | | |
| --- | --- | --- | --- | --- | --- | --- |
|  | **All Participants** | | **No CVD History** | | **CVD History** | |
| **Models** | **OR (95% CI)** | **P value** | **OR (95% CI)** | **P value** | **OR (95% CI)** | **P value** |
| **Model 1:** Rapid kidney function decline | 3.84 (3.01-4.89) | <0.0001 | 3.31 (2.27-4.82) | <0.0001 | 4.13 (2.48-4.84) | <0.0001 |
| **Model 2:** Model 1 **+** ACCORD trial treatment assignments and ACCORD clinical centers | 4.06 (3.16-5.20) | <0.0001 | 3.58 (2.43-5.27) | <0.0001 | 4.28 (3.02-5.91) | <0.0001 |
| **Model 3:** Model 2 + sex, age, duration of diabetes, BMI, WC, baseline HbA1c, SBP, DBP, HDL, log triglycerides, smoking history, diuretic therapy, beta blocker therapy, RASB therapy, CVD history at baseline (All participants), mean Hba1c during follow-up | 3.99 (3.00-5.30) | <0.0001 | 2.98 (1.93-4.62) | <0.0001 | 4.88 (3.31-7.12) | <0.0001 |
| **Model 4:** Model 3 + eGFR and log UACR at baseline | 3.36 (2.50-4.51) | <0.0001 | 2.58 (1.65-4.04) | <0.0001 | 4.17 (2.78-6.24) | <0.0001 |
| **Model 5:** Model 4 + last eGFR and log UACR before censoring or event | 4.90 (3.44-6.99) | <0.0001 | 3.76 (2.19-6.47) | <0.0001 | 6.20 (3.82-10.0) | <0.0001 |
| Data are based on the analysis of 4,758 participants who completed 5 years of follow-up and/or had an HF events within that time interval. Included were 832 participants with rapid kidney function decline and 303 participants with an HF event.  Abbreviations: OR, odds ratio; 95% CI, 95% confidence interval; BMI, body mass index; WC, waist circumference; HbA1c, glycated hemoglobin; SBP, systolic blood pressure; RASB, renin-angiotensin blockers; CVD, cardiovascular disease; eGFR, estimated glomerular filtration rate; UACR, urine albumin-creatinine ratio | | | | | | |

| **Supplementary Table 4. Odds of HF within 4 years from baseline according to baseline eGFR and UACR classes.** | | | | |
| --- | --- | --- | --- | --- |
| **Baseline eGFR**  **(ml/min/1.73 m^2^)** | **Baseline UACR**  **(mg/g)** | **No HF**  **N (%)** | **HF**  **N (%)** | **Adjusted**  **OR (95% CI)** |
| ≥90 | <30 | 2,452 (33.8) | 33 (13.0) | 1 (ref) |
| ≥90 | ≥30 | 1,009 (13.9) | 56 (22.1) | 3.51 (2.15-5.74) |
| 60-89 | <30 | 2,172 (30.0) | 46 (18.1) | 1.17 (0.71-1.94) |
| 60-89 | ≥30 | 948 (13.1) | 72 (28.4) | 3.53 (2.18-5.71) |
| <60 | <30 | 390 (5.4) | 20 (7.9) | 2.12 (1.11-4.03) |
| <60 | ≥30 | 280 (3.9) | 27 (10.6) | 3.98 (2.19-7.23) |
| All | All | 7,251 (100) | 255 (100) |  |
| Abbreviations: HF, heart failure; eGFR, estimated glomerular filtration rate; UACR, urinary albumin/creatinine ratio; OR, odds ratio; CI, confidence interval. | | | | |

# **Supplemental Figure 1. Measurements performed at baseline and follow-up.**

Randomization and start of follow-up

4 months

**UACR**

**eGFR**

**WATCH_DM**

**At least 3 eGFR measures or more were used to calculate the GRF slope**

**HF status after 4 years of follow-up**

# **Supplemental Figure 2. Flow chart for the selection of study participants**

Abbreviations**:** eGFR, estimated glomerular filtration rate; UACR, urinary albumin/creatinine ratio

**
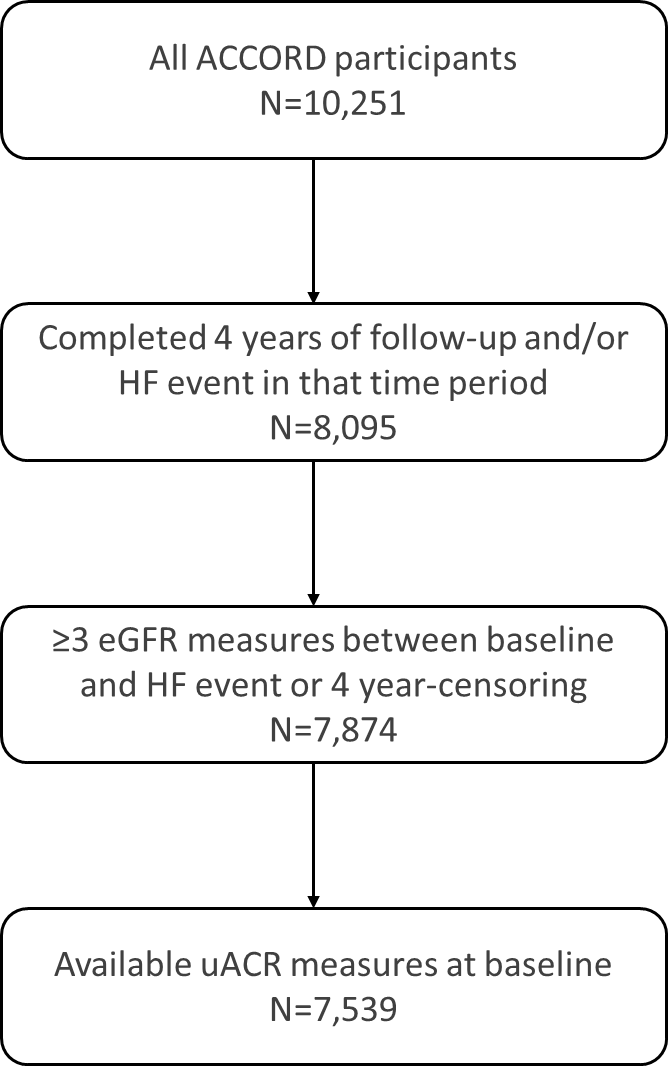
**

# **Supplemental Figure 3. Distributions of the number of eGFR measurements (A) and years spanned by them (B)**

|  |  |
| --- | --- |
| **A**  **B** |  |
| 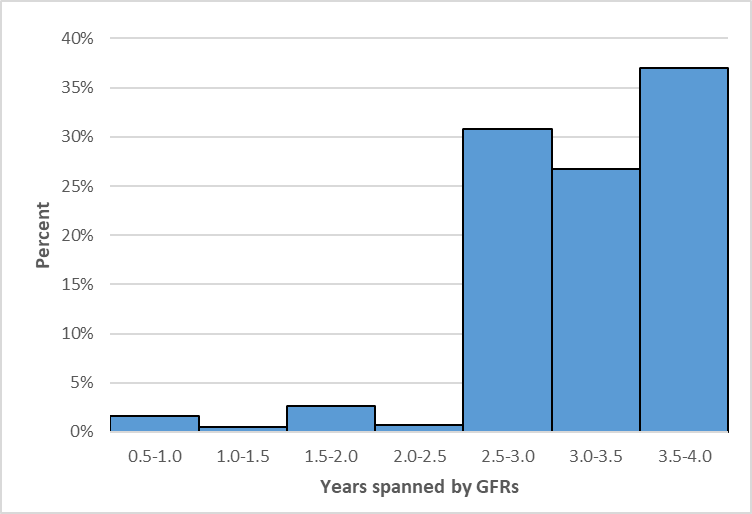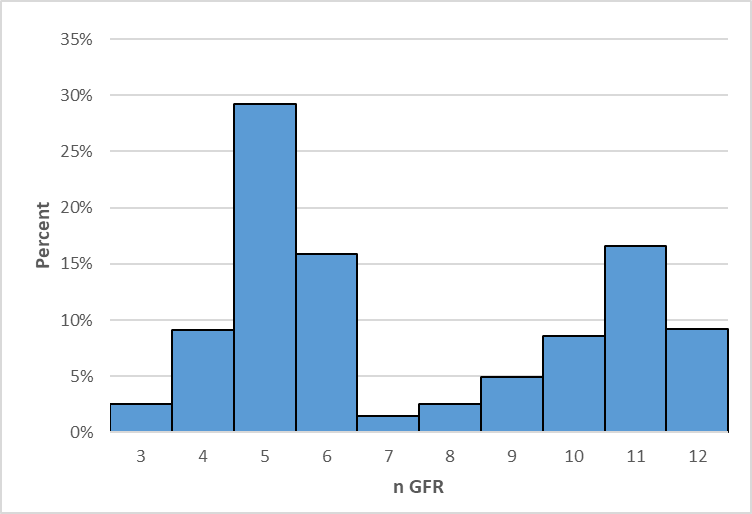 |  |
